# Design of a low-cost force insoles to estimate ground reaction forces during human gait

**DOI:** 10.1016/j.ohx.2024.e00589

**Published:** 2024-09-27

**Authors:** Nelson E. Guevara, Carlos F. Rengifo, Yamir H. Bolaños, Daniel A. Fernández, Wilson A. Sierra, Luis E. Rodríguez

**Affiliations:** a Research Group of Automation, Universidad del Cauca, Colombia; b Department of Electronic, Instrumentation and Control, Universidad del Cauca, Colombia; c Faculty of Engineering and Natural Science, Corporación Universitaria Autónoma del Cauca, Colombia; d Faculty of Biomedical Engineering, Escuela Colombiana de Ingeniería Julio Garavito, Colombia

**Keywords:** Ground reaction forces, Force insoles, Piezo-resistive sensors, ESP32 microcontrollers, Low-cost, 3D printing

## Abstract

This paper proposes a low-cost electronic system for estimating ground reaction forces (GRF) during human gait. The device consists of one master node and two slave nodes. The master node sends instructions to slave nodes that sample and store data from two force insoles located at the participant’s feet. These insoles are equipped with 14 piezo-resistive FlexiForce A301 sensors (FSR). The slave nodes are attached to the ankles and feet of each participant. Subsequently, the start command is transmitted through the master node, which is connected to the USB port of a personal computer (PC). Once the walking session is completed, the information obtained by the slave nodes can be downloaded by accessing the access point generated by these devices through Wi-Fi communication. The GRF estimation system was validated with force platforms (*BTS Bioengineering P6000, Italy*), giving on average a *fit* measure equal to 68.71%±4.80% in dynamic situations. Future versions of this device are expected to increase this *fit* by using machine learning models.

## Graphical abstract

**Figure:**
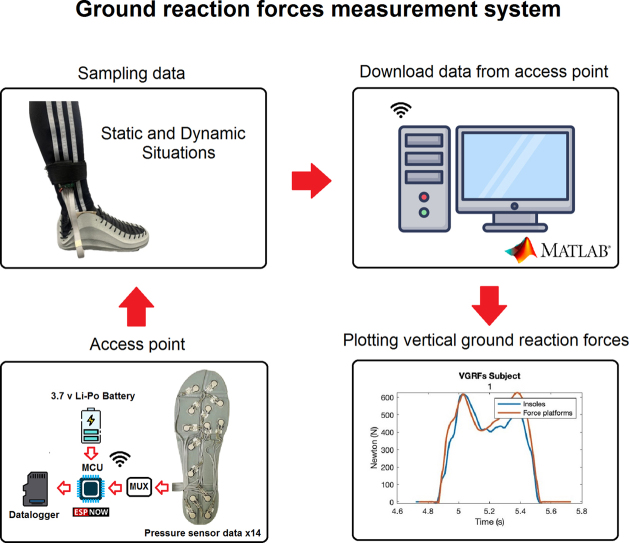

## Specifications table

**Table:** 

**Hardware name**	Ground Reaction Forces Measurement System (GRFMS)
**Subject area**	• Medical
**Hardware type**	• System for measurement of biomechanical variables
**Closest commercial analog**	No commercial analog is available.
**Open source license**	CC BY 4.0
**Cost of hardware**	$800 USD
**Source file repository**	http://doi.org/10.17632/rbyv2xx5dr.1

## 1 Hardware in context

The study of human gait is of great relevance in medicine [1] and engineering [2] since it is an essential ability to carry out daily activities; however, this ability deteriorates with aging [3]. This problem has motivated the development of motion capture systems such as optical, inertial, and force platforms, which generate inputs for quantitative gait analysis that provide measures of central tendency and dispersion of gait variables such as step length, step width, gait speed, joint range of motion, and ground reaction forces (GRF). In particular, the GRF are frequently measured because they help to detect alterations in the musculoskeletal system. Their importance lies in the fact that it is possible to identify neurodegenerative diseases, evaluate the efficiency of sports gestures, evaluate osteomuscular factors involved in lower limb injuries, and estimate the risk of falls. GRF can be estimated using force platforms and force insoles [4], [5], [6], [7], [8]. The former are intended for indoor use and limit the motion capture experiment to a few steps. On the contrary, force insoles can be used outdoors without any limitations on the number of steps. Both types of devices are expensive, approximately $20.000 USD and often provide only average values of the estimated parameters, which can result in a disadvantage in terms of profitability and data analysis.

This article focuses on the development of force insoles that we expect to use in later studies for fall risk assessment using the plantar center of pressure (CoP). Our main motivation is that currently fall risk assessment is based on subjective tests such as Performance Oriented Mobility Assessment (POMA) [9], [10], [11], Timed and Go (TUG) [12], [13], and Berg Balance Scale (BBS) [14], [15], whose results depend on the expertise of the person conducting the test. With the CoP, a mature and validated concept in bipedal robots community [16], [17], [18], we expect to propose objective fall risk assessment.

**Fig. 1:**
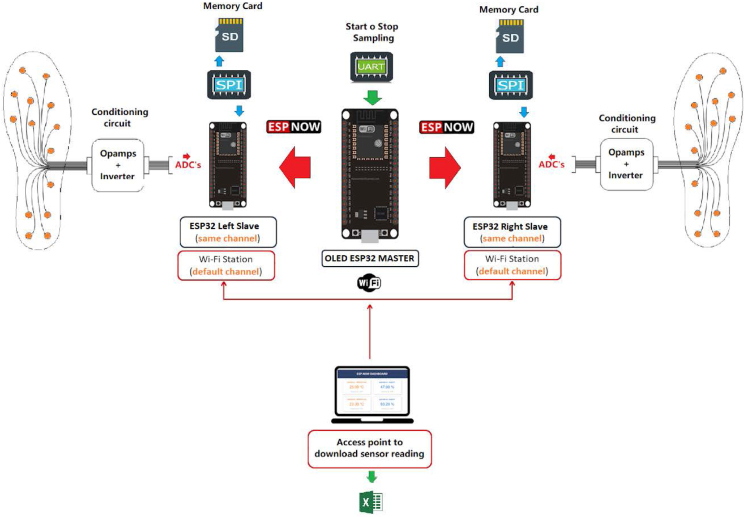
General scheme of a GRFMS.

During the last decade several force insoles have been proposed. In [19], the authors propose to simulate a force insoles with 16 piezo-resistive sensors using the data of 39 healthy adults. These data were obtained using a Pedar-X commercial system (Munich, Germany), which consists of a matrix of 99 sensors. The 16 contact points were established from the areas that received the most pressure during the walking phases using the Pedar-X system. To enhance the data set, the Pedar-X system was synchronized with a stereophotogrammetric system (6 cameras, 60 Hz) and a force platform (Bertec FP2060, 960 Hz). In [5], the authors present the design of a neural network to estimate GRF and CoP in an insole equipped with 6 piezo-resistive sensors. The prototype was validated using a force platform (F-Scan, TEKSCAN Inc. USA) and 8 healthy adults. The authors found that it is feasible to accurately and precisely estimate GRF and CoP with low-cost devices. However, the authors, unlike [4], [5], [20], [21], did not report how to replicate their proposed device. In this sense, the electronic system proposed in this article for the estimation of GRF is replicable, portable, low-cost, and low-energy consumption. The prototype described here comprises two main parts. One are the insoles equipped with 14 piezo-resistive sensors (FlexiForce A301, TEKSCAN Inc. USA), which measures the reaction forces between the foot and the ground during walking in people weighing between 50 and 80 kg. The other part is a desktop application built to process the forces measured by the insoles.

## 2 Hardware description

**Fig. 2:**
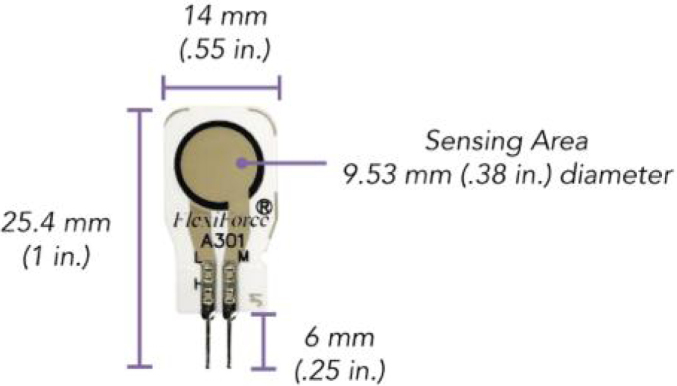
FlexiForce A301 sensor.

**Fig. 3:**
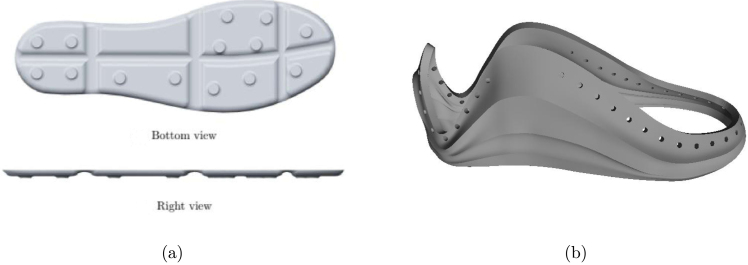
3D shoe design. (a) Insole design and (b) coatings design.

The proposed Ground Reaction Forces Measurement System (GRFMS), shown in Fig. 1, consists of the following elements: (i) a master node (OLED ESP32 Master) that receives instructions from a computer through the USB port and forwards these values to its slave nodes through the ESP-NOW wireless communication protocol; (ii) two slave nodes (ESP32 Left Slave and ESP32 Right Slave) that acting *Access Point*, and are responsible for sampling, conditioning, and storing data at a sampling frequency of 100 Hz. This is achieved through the analog-digital multiplexer 74HC4067, operational amplifiers MCP6004, and removable *flash* memories (SD); (iii) two insoles equipped with 14 FlexiForce A301 sensors each (see Fig. 2, Fig. 3).

### 2.1 Electronic design

**Fig. 4:**
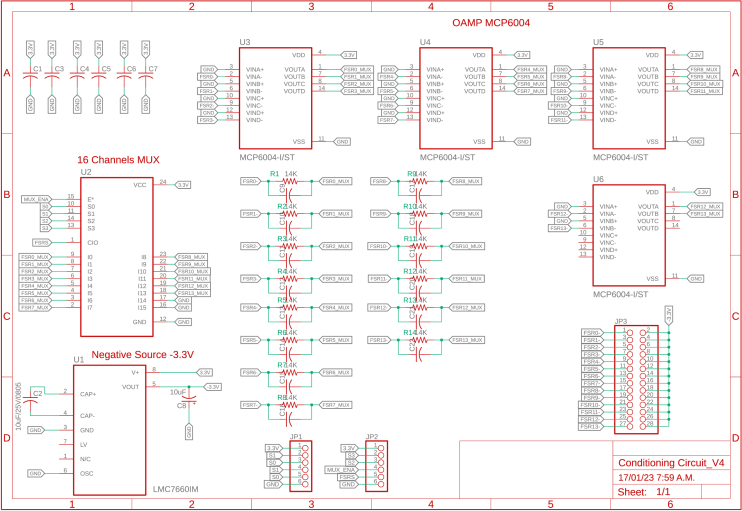
Conditioning circuit.

**Fig. 5:**
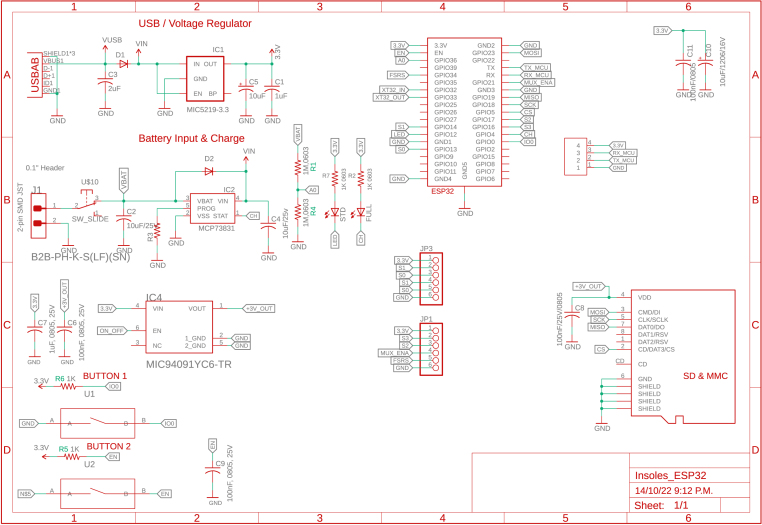
Control circuit.

**Fig. 6:**
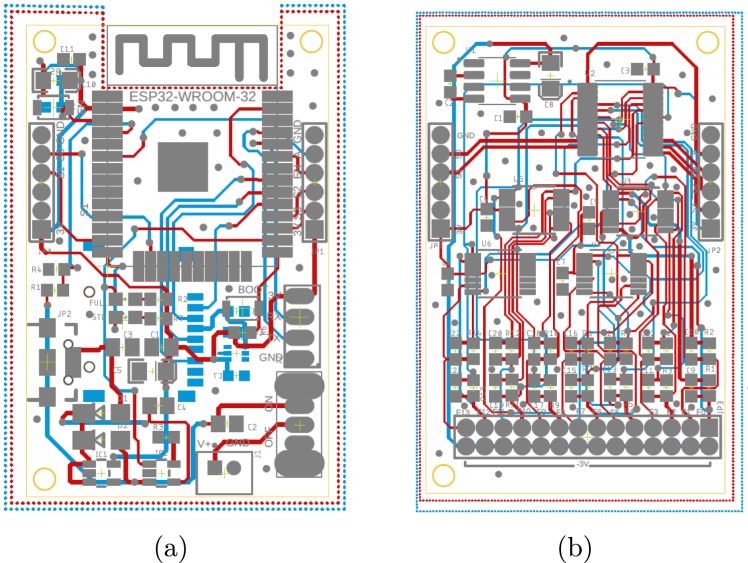
Print circuit boards. (a) Control PCB and (b) Conditioning PCB.

During the implementation phase of each slave node, a schematic interconnection diagram composed of two stages was created. The first corresponds to the control circuit (see Fig. 5) and the second to the signal conditioning circuit (see Fig. 4). The Eagle Version 9.6.2 software was used for the design of the printed circuit boards (PCB), resulting in two small-sized boards (32×50mm) presented in Fig. 6.

### 2.2 Force insole design

**Fig. 7:**
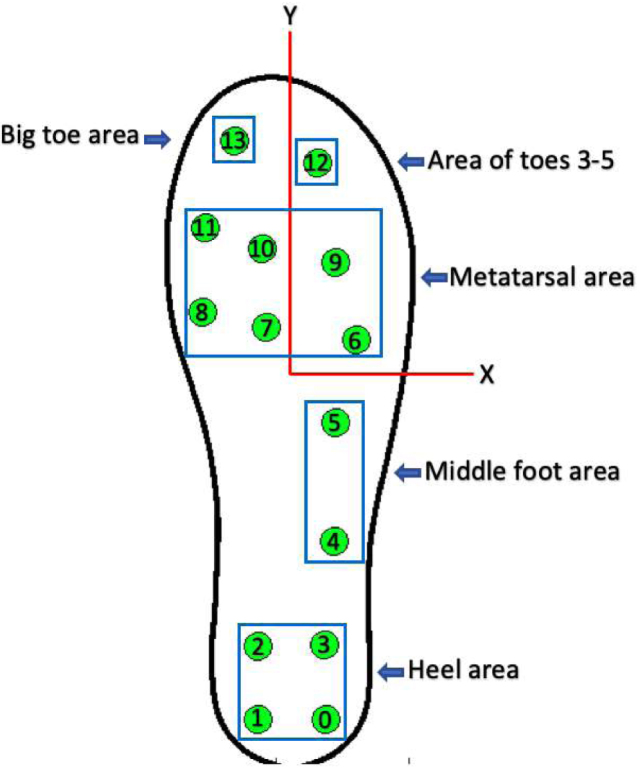
Distribution of sensors.

The location of sensors is an important factor in accurately estimating GRFs. Hence, the 14 FSR sensors were fixed according to the anatomical shape of the foot and its prominent areas, which experience greater pressure than the less prominent ones [8]. The distribution of sensors was carried out as follows: four sensors for the heel area, two sensors for the middle foot area, six sensors for the metatarsal area, one sensor for the big toe, and finally, one sensor for the area of toes three to five, as shown in the Fig. 7. The 3D model of the insoles and its sensor distribution was taken from [8], (see Fig. 3(a)); however, as there was no direct contact with the ground, the measured force was too low when inserting the insoles with the sensors inside the user’s shoes, this may be due to the flexible materials of the footwear, which allows deflections and angled areas of the sensor. To resolve this issue, two coatings were designed and manufactured that are fixed to each insole and must be used by all participants (see Figs. 3(b), 15). This approach ensures that the measured force is concentrated exclusively at the 14 contact points (FSRs). The manufacturing criteria of the insoles were as follows: (i) allow foot flexion without altering the gait pattern; (ii) being comfortable, lightweight and thin; (iii) adequately transmitting forces through the sensitive areas of the insole (pucks, see Fig. 12(a)); (iv) withstanding humidity and temperature conditions within the shoe in the range of 20 to 45°C. The Table 1 shows the coordinate system of each FSR sensor, while Fig. 7 illustrates its distribution on the insole.

**Table 1:** Coordinate system on the insole.

Sensor	X (mm)	Y (mm)
0	12.50	−128.49
1	−12.50	−128.49
2	12.50	−100.49
3	−12.50	−100.49
4	16.44	−61.13
5	16.80	−16.62
6	25.03	14.67
7	−8.41	19.48
8	−32.95	25.50
9	16.98	43.74
10	−10.36	49.02
11	−31.65	57.24
12	10.56	81.05
13	−20.74	89.47

**Fig. 8:**
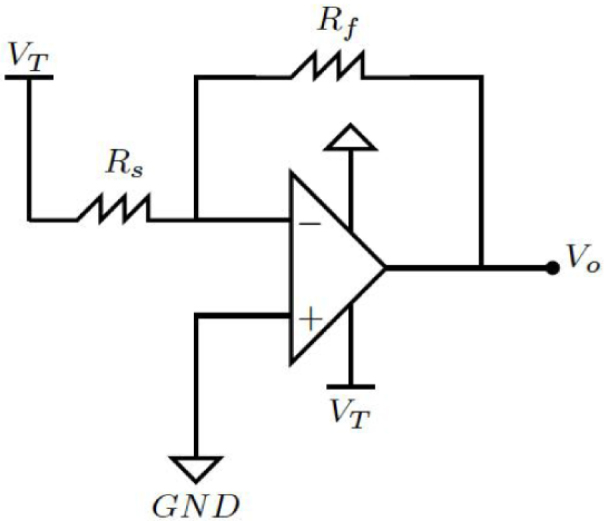
Recommended circuit for FlexiForce sensor. The operational amplifier is a MCP6004 of Microchip™.

### 2.3 FSRs calibration

The FSR were read through a circuit that converted resistance changes into voltage changes. A voltage divider was a viable alternative; however, the change in FSR resistance with respect to the applied force is not linear, while the change in conductance, the reciprocal property, is approximately linear according to the manufacturer [22]. An operational amplifier (MCP6004) was used in an inverting configuration to map changes in conductance to voltage changes (0 to 3.3,V) and finally relate this latter variable to the applied force. The circuit implemented for each FSR is shown in Fig. 8. At steady state, the output voltage (V) is described by Eq. (1):

(1) $$V_{o}=-V_{T}\;\frac{R_{f}}{R_{s}}$$

where $-V_{T}$ is the supply voltage (V), $R_{s}$ is the FSR resistance (Ω), which varies depending on the applied force, and $R_{f}$ is the reference resistance (Ω). If a participant is considered to weigh 75kg (≈750N) and the body weight is distributed among a maximum of 5 sensors in each gait phase, then each sensor would have to withstand up to 15kg (≈150N). This value is lower than the standard range of FlexiForce sensors, which can measure up to ≈440N. The value of $R_{f}$ was adjusted so that the output voltage was around 3.3V. This way, saturation is avoided, and the full voltage range is used, as indicated by the manufacturer [22]. The resulting resistance value of $R_{f}$ was 50kΩ. Additionally, the supply voltage (−3.3V) is obtained through the LMC7660 integrated circuit. As a result, the current flowing (A) through the sensor at full load (when $R_{f}=R_{s}$) is represented by Eq. (2), whose value is well below the maximum recommended current by the manufacturer of 2.5mA [22].

(2) $$I=\frac{3.3\;V}{50\;k\Omega }=0.066\;mA$$

**Fig. 9:**
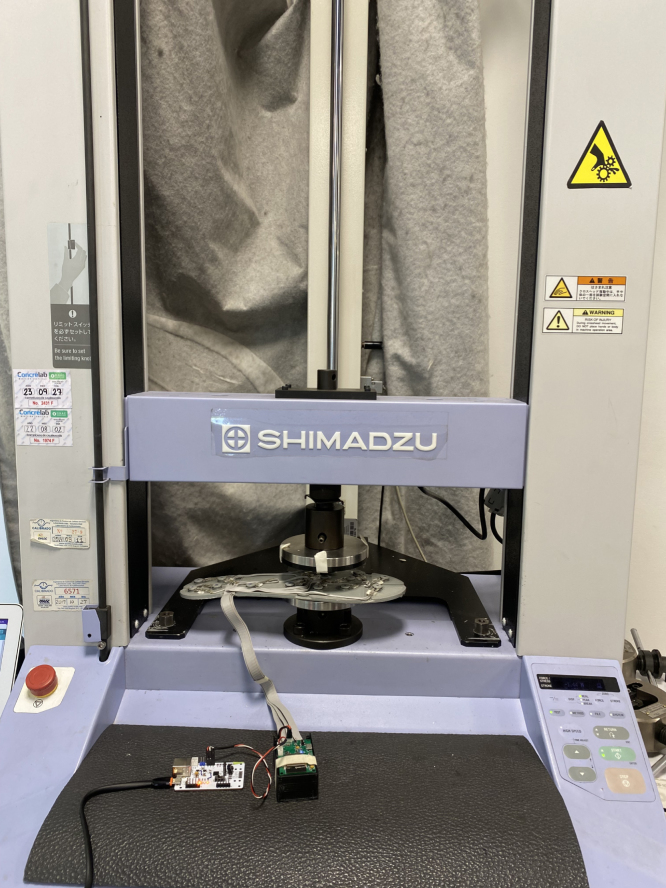
Shimadzu press.

**Fig. 10:**
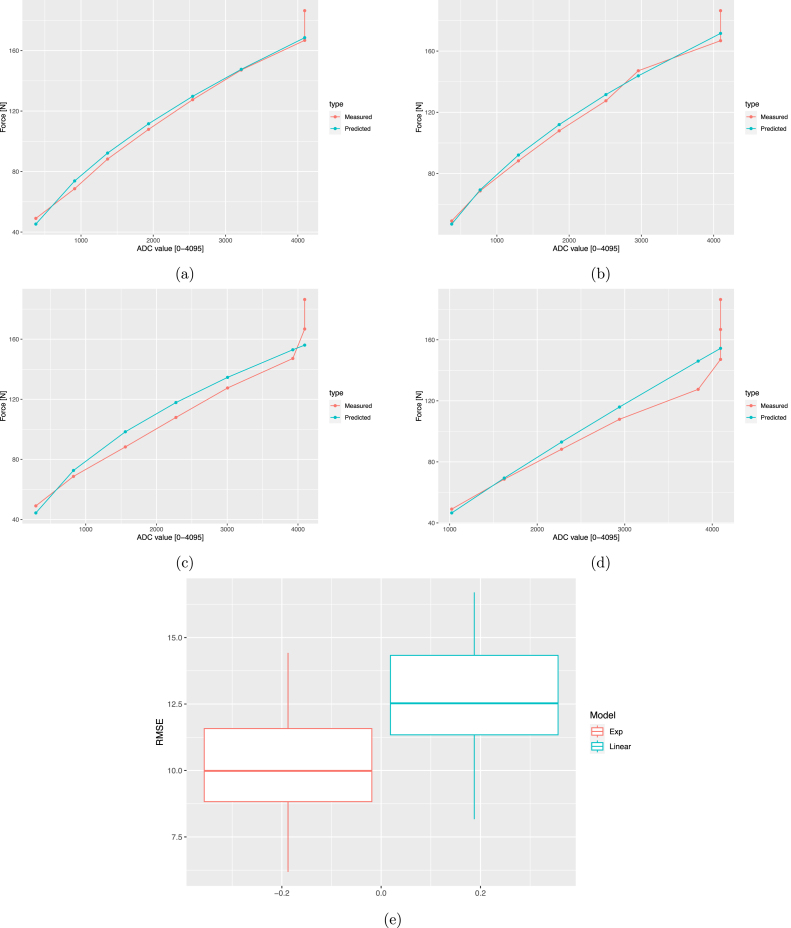
Statistical results. (a) FSR0’s exponential regression, (b) FSR9’s exponential regression, (c) FSR10’s exponential regression, (d) FSR13’s exponential regression and (e) Linear and Exponencial models.

Linear and exponential regressions were applied to each sensor and then compared via a paired Student’s T test to determine which of the two models leads to the lower root mean square error (RMSE). The 95% confidence interval for the difference in the RMSE mean values of the exponential and the linear models was [0.83,4.39]N, which is located on the positive semi-axis of the real line, indicating that, on average, the linear model exhibits a higher RMSE than the exponential model (p-value =0.007). The data for the regressions were obtained by applying eight weights of known value to each sensor (0, 5, 7, 9, 11, 13, 15, 17, 19kg), using a *Shimadzu press (Kyoto, Japan)* as shown in Fig. 9. The exponential regression for calculating the force (N) is described by Eq. (3):

(3) $$F=a\;ADC^{b}$$

where ADC is the numerical value delivered by the analog-to-digital converter (0−4095), and a and b are values determined through logarithmic regression (see Table 2). The exponential model has the advantage that ensures zero force when ADC is zero. On the contrary, the linear model (F=mADC+c) gives a non-null force when foot is not contacting ground due to the sum of the intercepts (c) of each sensor. Figs. 10(a), 10(b), 10(c), and 10(d) present the sensors with the best and worst fit on the left insole, while Fig. 10(e) shows the box plot for the RMSE values of the exponential and linear models.

The most relevant features of the electronic system are described below:

**Table 2:** Parameters of the 14 sensors of each insole.

Sensor	Right Insole			Left Insole		
	a	b	RMSE	a	b	RMSE
0	1.58	0.56	2.80	1.74	0.54	7.04
1	0.49	0.68	9.61	8.11	0.36	9.31
2	0.80	0.62	10.9	7.09	0.38	9.46
3	0.91	0.62	3.12	5.10	0.41	9.82
4	0.40	0.70	14.47	11.5	0.31	11.90
5	0.48	0.67	14.66	0.49	0.69	12.74
6	3.67	0.45	10.01	1.15	0.64	8.66
7	2.37	0.51	6.52	5.73	0.50	7.54
8	1.69	0.54	7.41	0.29	0.76	10.59
9	0.64	0.66	5.02	1.89	0.54	6.18
10	1.21	0.58	8.86	2.92	0.47	13.02
11	2.51	0.50	6.93	0.11	0.87	10.15
12	2.25	0.51	6.34	0.19	0.80	10.28
13	0.87	0.62	5.2	0.11	0.86	14.42

• GRFMS estimates GRFs using FlexiForce sensors, which are more accurate and less sensitive to noise than other piezo-resistive sensors.
• GRFMS is easy to install thanks to its ergonomic and sporty design.
• GRFMS is a portable and lightweight system, facilitating the transport and comfort of the device.
• GRFMS is a low-power system which data collection experiments comprising hundreds of steps.
• GRFMS stores the data captured by the FSR sensors in comma-separated value (CSV) format for further analysis.

## *Design files*

The design files are available at: http://doi.org/10.17632/rbyv2xx5dr.1

## 3 Design files summary

**Table:** 

Design filename	File type	Open source license	Location of the file
Right_Sneaker	CAD	CC BY 4.0	http://doi.org/10.17632/rbyv2xx5dr.1
Left_Sneaker	CAD	CC BY 4.0	http://doi.org/10.17632/rbyv2xx5dr.1
Lower left insole	CAD	CC BY 4.0	http://doi.org/10.17632/rbyv2xx5dr.1
Lower right insole	CAD	CC BY 4.0	http://doi.org/10.17632/rbyv2xx5dr.1
Top left insole	CAD	CC BY 4.0	http://doi.org/10.17632/rbyv2xx5dr.1
Top right insole	CAD	CC BY 4.0	http://doi.org/10.17632/rbyv2xx5dr.1
Case	CAD	CC BY 4.0	http://doi.org/10.17632/rbyv2xx5dr.1
Heltec_case_-_Bottom	CAD	CC BY 4.0	http://doi.org/10.17632/rbyv2xx5dr.1
Heltec_case_-_Top	CAD	CC BY 4.0	http://doi.org/10.17632/rbyv2xx5dr.1

All 3D printable parts were built with Autodesk Fusion 360 and Solid Edge. STL files are provided for these parts. The printing material and the number of parts to be printed are shown in Table 3.

**Table 3:** 3D printable parts for the GRFMS.

Part	Material	Number	Support
Right_Sneaker	TPU (A90)	1	Yes
Left_Sneaker	TPU (A90)	1	Yes
Lower left insole	TPU (A90)	1	Yes
Lower right insole	TPU (A90)	1	Yes
Top left insole	TPU (A90)	1	Yes
Top right insole	TPU (A90)	1	Yes
Case	PLA	2	No
Heltec_case_-_Bottom	PLA	1	No
Heltec_case_-_Top	PLA	1	No

## *Bill of materials*

The bill of materials can be found at: http://doi.org/10.17632/rbyv2xx5dr.1

## 4 Bill of materials summary

The following table shows the materials used to design the GRFMS.

**Table:** 

Designator	Component	Number	Cost per unit (USD)	Total cost (USD)	Source of materials	Material type
1	ESP32 VROOM 160 MHz	2	$ 7.97	$ 15.94	Didácticas electrónicas	Other

2	OLED ESP32 VROOM 160 MHz	1	$ 11.64	$ 11.64	Mercado libre	Other

3	Capacitor 1 μF C0805 SMD	2	$ 0.17	$ 0.34	Digikey	Other

4	Capacitor 10 μF/25 V C0805 SMD	4	$ 0.17	$ 0.68	Digikey	Other

5	Capacitor 10 μF/16 V EIA3216 SMD	6	$ 0.17	$ 1.02	Digikey	Other

6	Capacitor 100 nF/25 V C0805 SMD	8	$ 0.17	$ 1.36	Digikey	Other

7	Capacitor 1 μF/25 V C0603 SMD	2	$ 0.17	$ 0.34	Digikey	Other

8	Capacitor 10 μF/25 V C0603 SMD	42	$ 0.07	$ 2.94	Digikey	Other

9	Diode DO214AC	4	$ 0.19	$ 0.76	Digikey	Other

10	Regulator MIC5219-3.3 SOT23-5 SMD	2	$ 0.48	$ 0.96	Digikey	Other

11	Controller MCP73831 SOT23-5 SMD	2	$ 0.76	$ 1.52	Digikey	Other

12	Switch MIC94091Y-C6 1CH-5.5 V-1.2 A SMD	2	$ 0.42	$ 0.84	Digikey	Other

13	Connector 2 Positions Header (2 mm)	2	$ 0.21	$ 0.42	Digikey	Other

14	Connector 2.2MM CONNECTOR 4PIN	2	$ 0.21	$ 0.42	Digikey	Other

15	Pin header 1X06M	10	$ 0.083	$ 0.83	Digikey	Other

16	Connector USB-AB-MICRO-SMD-V03	2	$ 0.98	$ 1.96	Digikey	Other

17	Resistor 1M R0603	4	$ 0.1	$ 0.4	Digikey	Other

18	Resistor 1K R0603	8	$ 0.1	$ 0.8	Digikey	Other

19	Resistor 14K R0603	28	$ 0.1	$ 2.8	Digikey	Other

20	Holder MICROSD C12075	2	$ 1.98	$ 3.96	Digikey	Other

21	Switch SW SLDE	2	$ 0.5	$ 1	Digikey	Other

22	Converter LMC7660IM 1.5 V–10 V SOIC-8	2	$1.17	$ 2.34	Digikey	Other

23	Multiplexer CD74HC40-67SM96E4 SSOP-24	2	$ 0.92	$ 1.84	Digikey	Other

24	Amplifier MCP6004-I/ST SOP65P640X-120-14N	8	$ 0.57	$ 4.56	Digikey	Other

25	FlexiForce A301 Sensor	28	$ 23.3	$ 652	Tekscan	Other

The total cost of all components is $711.67 USD.

## 5 Build instructions

A service specialized in the design and manufacture of electronic systems carried out the production of two control PCBs (*Board Production Files/ESP32 Board*) and two signal conditioning PCBs (*Board Production Files/FSR Board*). Companies such as PCBWay™ or JLCPVB™ use BOM and Gerber files to perform this task. The documentation needed to replicate the electronic prototype is available in the public access repository: http://doi.org/10.17632/rbyv2xx5dr.1.

**Fig. 11:**
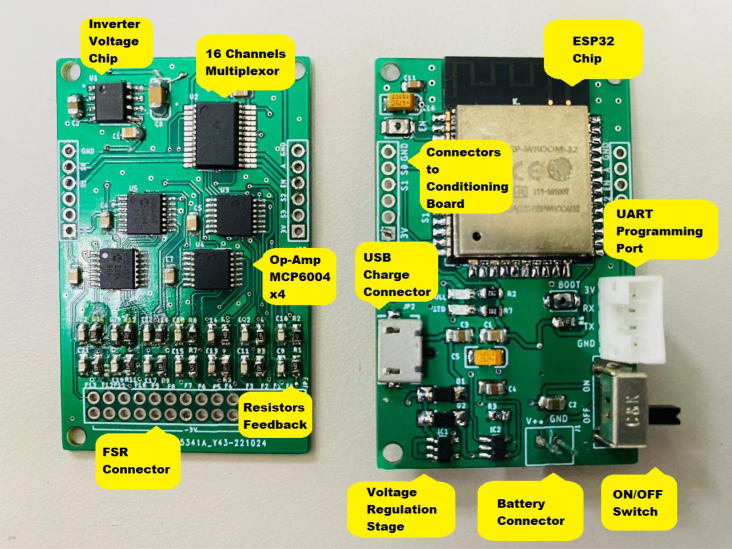
Electronics components.

**Fig. 12:**
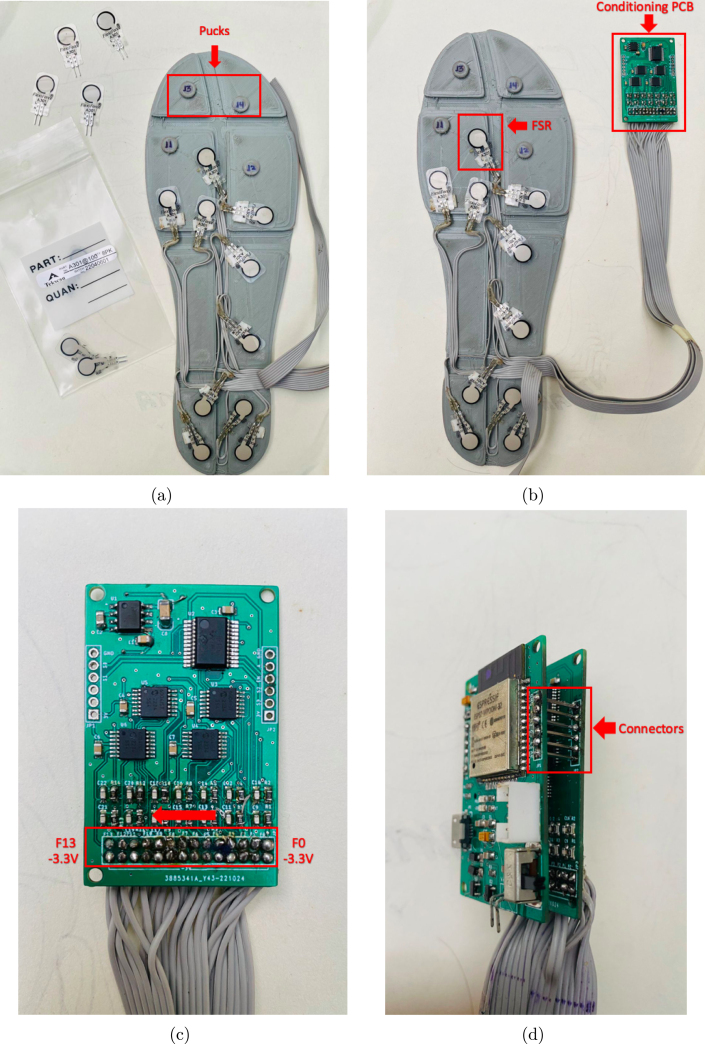
Sensor Connections. (a) Sensor attachment, (b) Connection of the sensors to the conditioning PCB, (c) Labels on conditioning PCB and (d) Connection between Control PCB and Conditioning PCB.

**Fig. 13:**
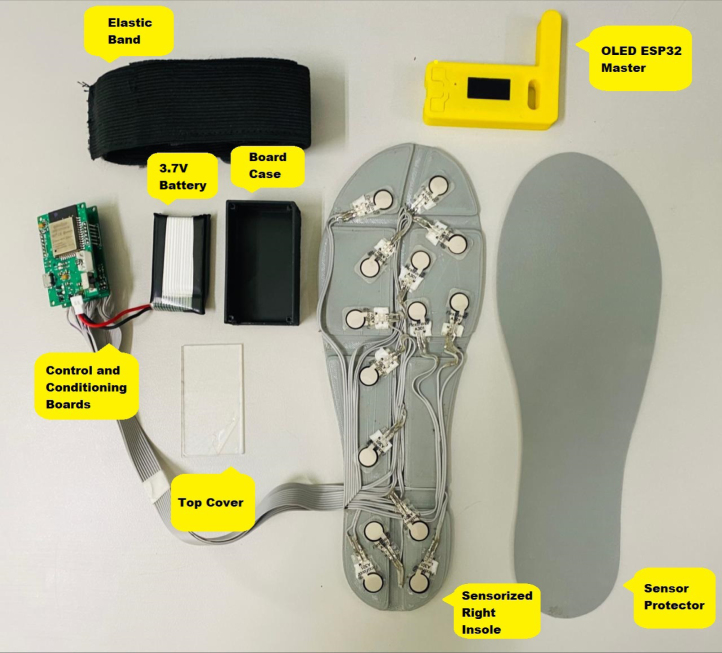
Components of a slave node without coating.

**Fig. 14:**
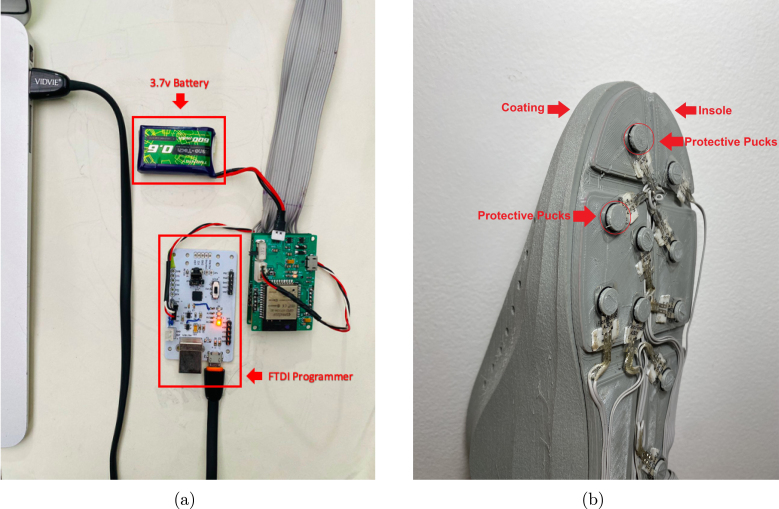
(a) Programming control PCB and (b) Coating and insole.

**Fig. 15:**
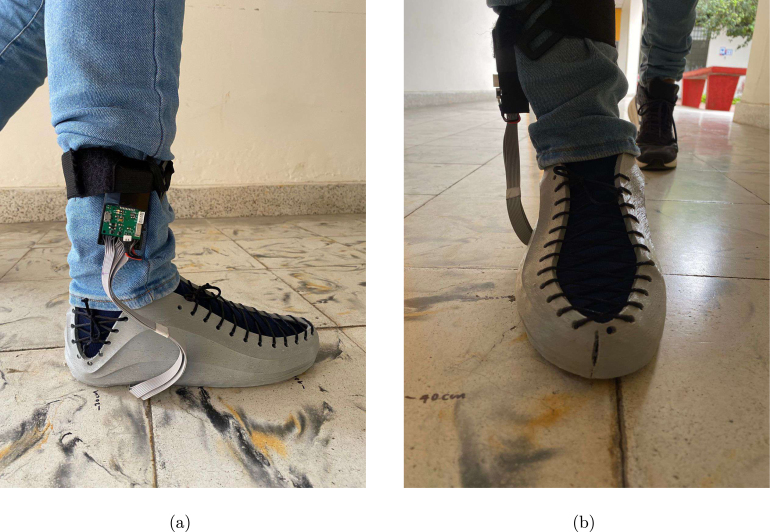
GRFMS prototype. (a) Lateral view and (b) Frontal view.

**Fig. 16:**
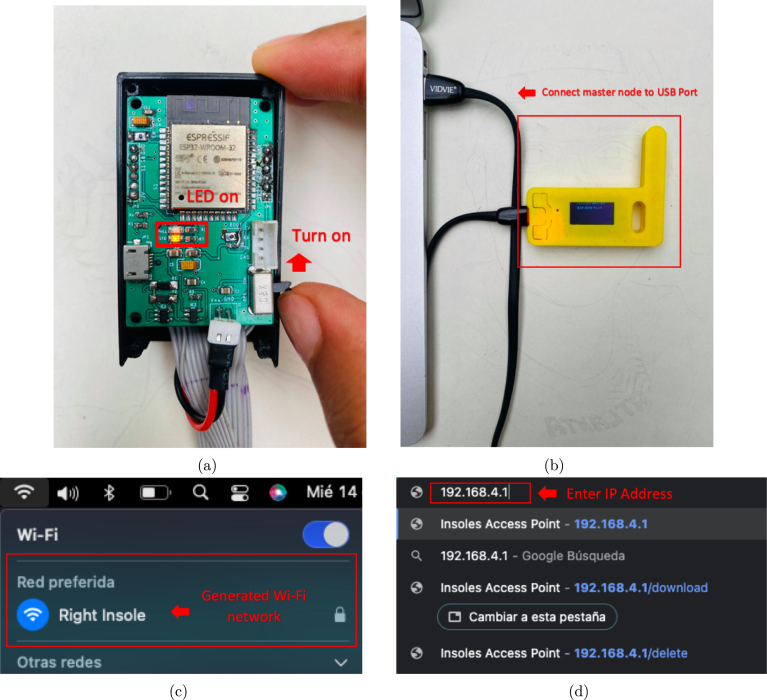
GRFMS configuration. (a) Turn on slave node, (b) Connect node master to USB port, (c) Generated Wi-Fi network and (d) Enter IP address.

The Fig. 13 shows an assembled slave node, which is ready to be bonded to its coating and tested in the laboratory. The hardware/software integration procedure is described below:

• The first step consisted of attaching each electronic component to the control and conditioning boards (see Fig. 11). This task is performed manually or by using SMD soldering ovens. The layout of the components on the printed circuit board can be found in the folder (*Board Production Files*) of the repository.
• The next step consisted of fabricating the insoles and their coatings using a 3D printer (see Fig. 3(b)). To achieve the desired level of flexibility and durability, it is recommended to use a Thermoplastic Polyurethane (TPU) filament with a hardness of A90 and a filler percentage greater than 90%. These specifications are commonly used in the manufacture of shoe soles. The STL files needed to perform this task can be found in the folder (*3D Insole Design*) of the repository.
• After printing the insoles and their coatings, 14 FlexiForce A301 sensors per insole were centered and attached using flexible glue, as shown in Fig. 12(a).
• Next, using ribbon cable (60 cm × 28 wires) and tin solder, each FSR sensor was connected by soldering one pin to −3.3 V and the other pin to its respective connector (F0 through F13) on the signal conditioning board, as shown in Fig. 12, Fig. 12. The connection order of the sensors is described in Fig. 7.
• After correctly connecting each sensor, the control and signal conditioning boards are soldered together using connectors (pin header 2.54 mm), fixing one board on top of the other, as shown in Fig. 12(d).
• Connect the battery (3.7 V and 600 mAh) and turning on the control board using the switch located on the right side (see Fig. 16(a)). Finally, the firmwares were loaded in the slave nodes and in the master node, respectively. For this task, it was necessary to connect each node to the USB port of the computer using an FTDI programmer and the open source Arduino IDE application, as shown in Fig. 14(a). The firmware developed for the slave nodes and master node can be found in the folders (*ESP32 Right Slave Firmware, ESP32 Left Slave Firmware and OLED ESP32 Master Firmware*) of the repository.
• Calibrate each sensor (28 units) following the procedure in Section 2.3 of the document.
• After calibrating each sensor, use 3D printing and laser cutting to manufacture cases and top covers for the master node and the slave nodes. These cases allow each slave node to be attached to the participant’s ankle using elastic bands (see Fig. 15, Fig. 13). The necessary files for this task are located in the (*Part For Enclosure CAD Files*) folder of the repository.
• Fix the insoles and their coatings with flexible glue, as shown in Fig. 14(b).
• Finally, fix the protective pucks to each sensor with flexible glue; this will prevent premature degradation of the FSR sensor due to direct friction with the ground during the walk test, as shown in Fig. 14(b). The protective pucks are made of the same material as the insoles and have the following dimensions: diameter of 9.53mm and thickness of 2mm, respectively.
• The maximum current to be supplied by the battery is calculated from the sum of the consumption of each electronic element. Table 4 shows these values.
  Referring to Table 4, a Li-Po type battery of 3.7V and 600mAh capacity will enable the electronic system to operate for:
  (4) $$\frac{600\;mAh}{123\;mA}=4.86\;h$$
  We recall that each node has its won battery.

**Table 4:** Approximate consumption of each sensor node.

Device	Consumption (mA)
ESP32	100
FlexiForce sensors x14	3
SD memory	20
Voltage regulator	0.5
**Total**	**123.5**

## 6 Operation instructions

**Fig. 17:**
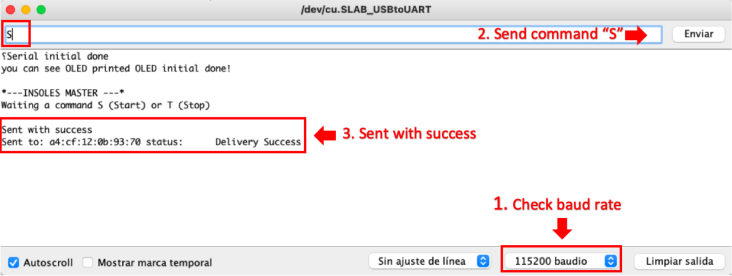
Serial communication parameters.

**Fig. 18:**
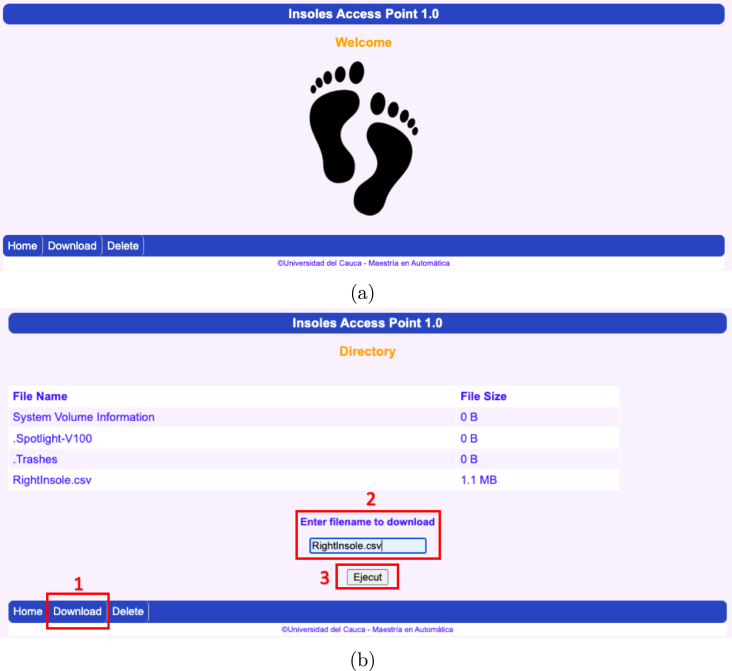
Web interface. (a) Home section and (b) Download section.

**Fig. 19:**
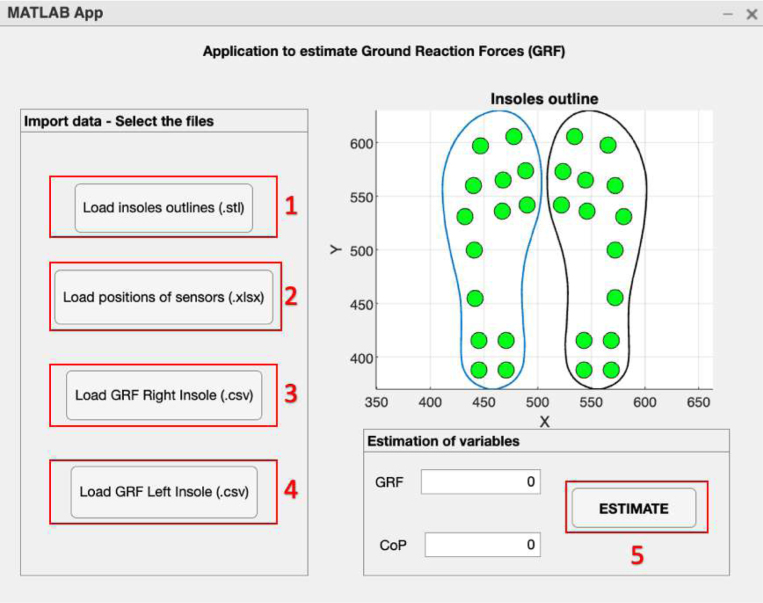
MATLAB desktop application.

After the hardware and software integration procedure, the prototype is attached to the participant’s feet and ankles using elastic bands (see Fig. 15, Fig. 15). The measurements are obtained by following the procedure described below:

• Power on the ESP32 Left and ESP32 Right slave nodes by changing the position of the switch on each node until an LED diode lights up, as shown in Fig. 16(a).
• Connect the OLED ESP32 master node to the computer via the USB port (Fig. 16(b)) and open the Arduino IDE desktop application. The default serial communication parameters are Baud Rate: 115,200 bps; Parity: none; Number of bits per character: 8 bits (see Fig. 17).
• To start the data capture, the person supervising the test sends the command *“S”* through Arduino IDE, which communicates with the master node via the serial port. If the command sent successfully, the following message will appear on the master node’s serial terminal: “*Sent with success*” (see Fig. 17).
• Once the data capture has been initiated, the participant should begin walking at a comfortable pace and cover a distance of 10 meters in a straight line. Subsequently, the participant should turn around and walk back to the starting point. It is important to maintain a natural gait and avoid altering the way one would normally walk (see Fig. 15).
• When the person supervising the test ends it by sending the command *“T”* through Arduino IDE. Subsequently, the slave nodes automatically act as a *Access Points*, generating the Wi-Fi networks *Right Insole* and *Left Insole*.
• To download data, the user must connect separately to each Wi-Fi network (*Right Insole* or *Left Insole*), as shown in Fig. 16(c). No password required.
• Once connected to the desired Wi-Fi network, the user must enter the IP address *192.168.4.1* in a web browser (see Fig. 16(d)). This will display the following web application (see Fig. 18(a)).
• The user should then select the *Download* tab of the web application (see Fig. 18(b)). This will list the (CSV) files stored in the SD memory of the slave node.
• In the text box presented in Fig. 18(b), the user should enter the name of the file they wish to download, either *RightInsole.csv* or *LeftInsole.csv*, depending on which slave node they are downloading data from. Then, they should press the *Execute* button to start the download.
• After downloading the *RightInsole.csv* and *LeftInsole.csv* files, the user should open the MATLAB desktop application and load the following files: 3D_Insole.stl, pos_sensors.xlsx, RightInsole.cvs and LeftInsole.csv, as shown in Fig. 19. The first two required files are located in the folder (*Matlab Desktop Application*) of the repository.
• Finally, press the *Estimate* button on the MATLAB desktop application to estimate the participant’s GRF.

The following are the usage recommendations:

1. Ensure that the batteries of the slave nodes are charged.
2. Attach the slave nodes properly to the participant’s ankle.
3. Download CSV files from each slave node separately.
4. Use Arduino IDE to send commands via the serial port.
5. Keep the master node within 30 meters of the slave nodes.

## 7 Validation and characterization

**Fig. 20:**
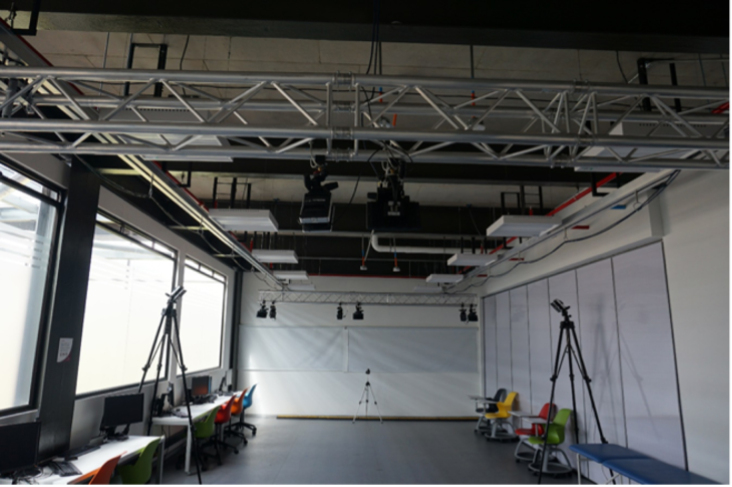
Motion analysis laboratory. Escuela Colombiana de Ingeniería Julio Garavito.

**Fig. 21:**
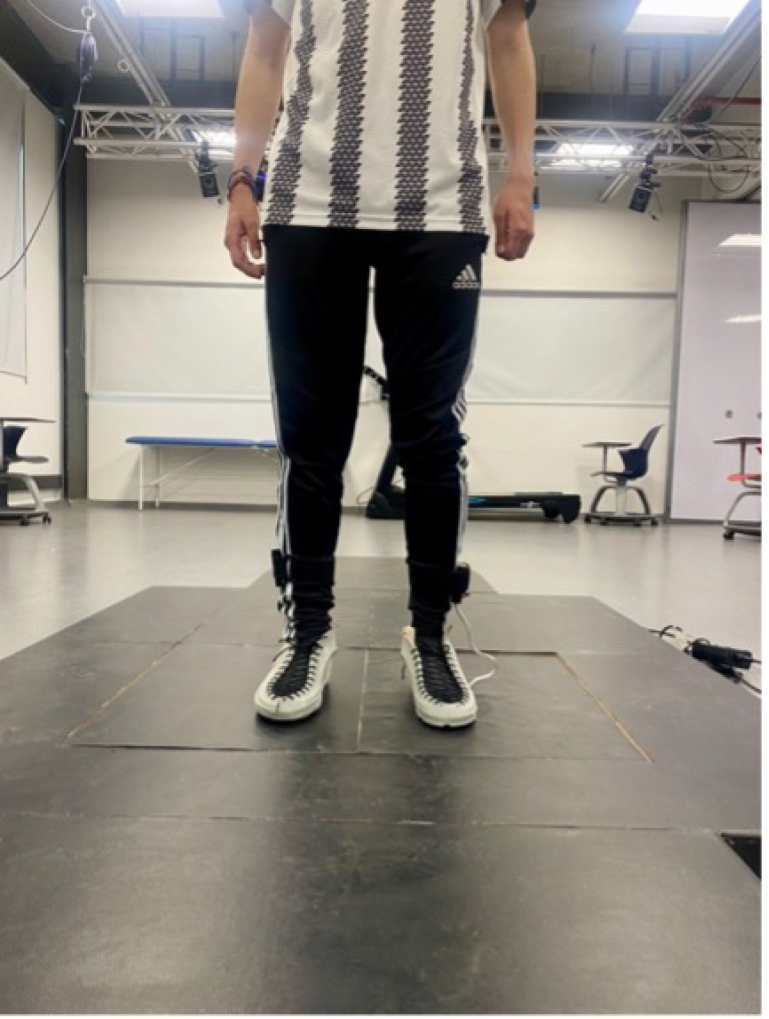
Subject 1 equipped with force insoles.

During the validation phase, the prototype was tested using two force platforms (*BTS Bioengineering P6000, Italy*) at the Motion Analysis Laboratory of the Escuela Colombiana de Ingeniería Julio Garavito in Bogotá D.C., Colombia, as shown in Fig. 20. To carry out the tests, four participants of both genders, with no history of pathologies on gait, were selected. Table 5 shows the demographic information of the subjects. Each subject gave informed consent and was equipped with the force insoles, as shown in Fig. 21.

The synchronization between the commercial equipment and the developed prototype was carried out by means of the *BTS Trigger Box* device, establishing a sampling frequency of 100 Hz in both devices. Measurements were performed as follows:

**Table 5:** Subject’s demographic information.

Subject	Age (years)	Height (m)	Mass (kg)	Foot size (US)	Gender
1	21	1.68	58	7.5	Male
2	21	1.68	62	8	Female
3	31	1.64	75	8	Female
4	22	1.64	61	8	Male

**Fig. 22:**
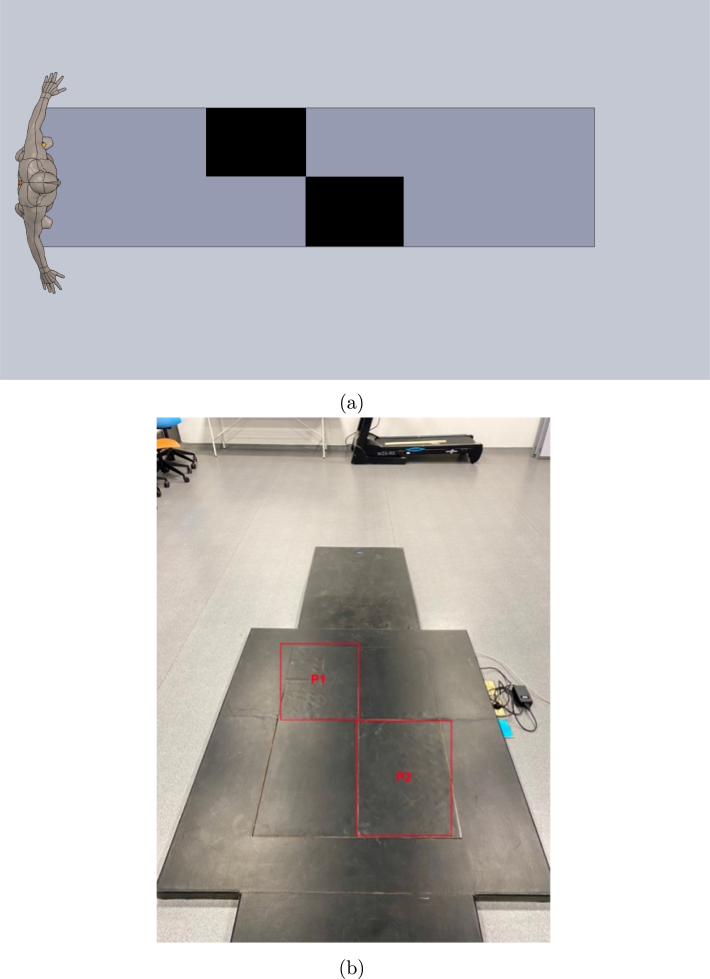
Distribution of platforms of force in the laboratory. (a) Diagram of force platform distribution and (b) Force platforms.

**Gait:** A 10-m walk in a straight line was performed, ensuring the contact of each foot with one force platform (see Fig. 22, Fig. 22). The participants were instructed to maintain a natural gait and avoid altering their usual gait.

**Fig. 23:**
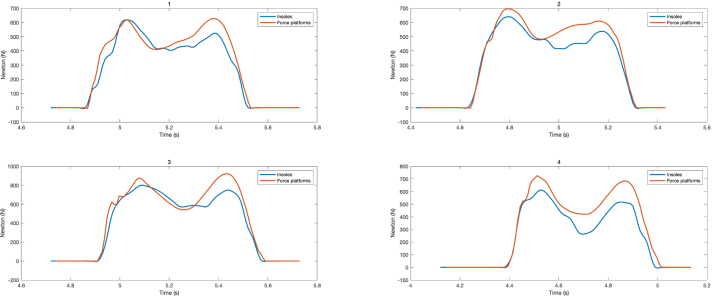
GRF of participants 1, 2, 3 and 4.

The GRFs magnitude was estimated by summing the forces measured by each FSR sensor using Eq. (5).

(5) $$GRF=\overset{n}{\underset{i=1}{\sum }}F_{i}$$

where $F_{i}$ is the force measured (N) by sensor i and n the number of sensors. The Fig. 23 shows the recording of the GRF as a function of time for each subject during the experiment. The figure illustrates a notable correspondence between the measurements obtained from the insole and the force platform. Additionally, the force profile characteristic of the human gait cycle is clearly observed. To evaluate the difference between the force vectors generated by the developed prototype and the commercial system for each participant during the gait test, a fit measure was calculated, which is described by Eq. (6):

(6) $$\text{fit}=\frac{\left\|x_{\text{ref}}-x\right\|}{\left\|x_{\text{ref}}-\text{mean}\left(x_{\text{ref}}\right)\right\|}$$

where xref (N) is the reference force vector (force platforms), x (N) is the corresponding force vector (force insoles), mean(xref) is the mean of the elements of the reference force vector and ‖⋅‖ represents the Euclidean norm of a vector. Thus, a higher value of the *fit* indicates a high similarity between the compared vectors. The results of this procedure are detailed in Table 6.

Analysis of the data (see Fig. 23 and Table 6) revealed that the force measured by the sensors was lower than the participant’s weight during locomotion; however, the *fit* for this first version of force insoles is relatively good. This discrepancy could be attributed to the drift of sensors caused by their use over time [8], the absence of sensors in some areas of the insole, the elasticity of the TPU, or various deformations. Therefore, in a second version of the prototype, this error is expected to be mitigated by recalibrating the sensors, adding more sensors and applying machine learning models. This approach follows the same procedure that has been used in previous studies such as [4], [5], which seek to improve the accuracy and precision in the estimation of GRFs [4].

**Table 6:** Estimated *fit* for each participant.

Subject	*fit*
1	69.26%
2	73.82%
3	69.54%
4	62.22%

In this study, the design and development of a low-cost electronic system that estimates the reaction forces generated during walking from the contact between the feet and the ground is presented. This system uses piezo-resistive sensors strategically located in the plantar area. In addition, the insoles and coatings of the system were manufactured with flexible materials such as TPU using 3D printers, which provides significant advantages in terms of ease of manufacturing, customization and low printing costs. Importantly, the number of sensors and their position can be optimized for each user, taking into account the size and anatomy of the foot, and characterization of each foot-resistive sensor in the insole, as a function of the force applied, revealed differences in sensitivity. These differences may be due to the position of the sensor or its deflections. Therefore, it is determined that each sensor is unique and must be calibrated separately. The system was validated in dynamic tests, where the user walked for 10 m. The results showed a good correspondence with a relatively good *fit* equal to 68.71%±4.80%. Therefore, having a system with the ability to estimate reaction forces represents a significant step forward that could bring benefits at both regional and national levels. This takes on special importance when considering the lack of equipment to perform diagnostics of this type, a deficiency that currently affects physiotherapists, geriatricians and orthopedists. Additionally, the prototype has improved the physical infrastructure of the laboratory of the Faculty of Electronics and Telecommunications of the Universidad del Cauca by allowing computerized measurement of gait-related variables.

The initial prototype showed promising results; nonetheless, for the second version of the device, we will consider to develop flexible printed circuit boards that can be affixed to the insole. This can be done to improve aesthetics, portability, and interconnection between piezoresistive sensors and signals conditioning boards. In addition, proof-of-concept tests using Velostat, a low-cost pressure-sensitive conductive material, are proposed as an alternative to replace the FlexiForce sensors.

The proposed GRFMS estimates GRFs with a *fit* equal to 68.71%±4.80%. With respect to the gait-mode experiments, the graphs exhibit quasi-periodic signals typical of a normal gait pattern (see Fig. 23). Therefore, this initial prototype becomes a promising alternative for the estimation of gait variables. Subsequently, it is expected to develop a segmentation algorithm to identify the phases of gait and estimate the stability of a person based on the center of plantar pressure (CoP).

Finally, it is important to note that, although we use the 3D design of the force insole reported in Ref. [8], our prototype presents significant improvements in the electronic design. These improvements include the creation of a Mesh network for communication between the master node and the slave nodes (see Fig. 1), the incorporation of a Wi-Fi access point for wireless data download from any mobile device or computer, as well as the design and implementation of a compact printed circuit board (PCB) that integrates the acquisition, signal conditioning and control stages, using an ESP32 development board. This integration made it possible to reduce size, weight and energy consumption, contributing to the creation of a lightweight and comfortable prototype that procure not alter the participant’s walking pattern during the test.

## Ethics statements

The experiments described in this work have been carried out in accordance with The Code of Ethics of the World Medical Association (Declaration of Helsinki) for experiments involving humans. In addition, the participants gave the informed consent approved by the Ethics Committee of the Universidad del Cauca.

## CRediT authorship contribution statement

**Nelson E. Guevara:** Validation, Software, Conceptualization. **Carlos F. Rengifo:** Writing – original draft, Data curation. **Yamir H. Bolaños:** Resources, Methodology. **Daniel A. Fernández:** Validation. **Wilson A. Sierra:** Validation. **Luis E. Rodríguez:** Writing – review & editing.

## Declaration of Generative AI and AI-assisted technologies in the writing process

During the preparation of this work the authors used ChatGPT to improve the clarity, fluency, and correctness of the manuscript. After using these tools/services, the authors reviewed and edited the content as needed and take full responsibility for the content of the publication.
